# Application of Seq2Seq Models on Code Correction

**DOI:** 10.3389/frai.2021.590215

**Published:** 2021-03-19

**Authors:** Shan Huang, Xiao Zhou, Sang Chin

**Affiliations:** ^1^ Department of Physics, Boston University, Boston, MA, United States; ^2^ Department of Computer Science, Boston University, Boston, MA, United States; ^3^ Department of Brain and Cognitive Science, Massachusetts Institute of Technology, Boston, MA, United States; ^4^ Center of Mathematical Sciences and Applications, Harvard University, Boston, MA, United States

**Keywords:** programming language correction, seq2seq architecture, pyramid encoder, attention mechanism, transfer learning

## Abstract

We apply various seq2seq models on programming language correction tasks on Juliet Test Suite for C/C++ and Java of Software Assurance Reference Datasets and achieve 75% (for C/C++) and 56% (for Java) repair rates on these tasks. We introduce pyramid encoder in these seq2seq models, which significantly increases the computational efficiency and memory efficiency, while achieving similar repair rate to their nonpyramid counterparts. We successfully carry out error type classification task on ITC benchmark examples (with only 685 code instances) using transfer learning with models pretrained on Juliet Test Suite, pointing out a novel way of processing small programming language datasets.

## 1 Introduction

Programming language correction (PLC), which can provide suggestions for people to debug code, identify potential flaws in a program, and help programmers to improve their coding skills, has been an important topic in the Natural Language Processing (NLP) area. Generally, code errors consist of two categories: one is explicit, syntax errors, and the other is implicit, logic errors that could cause failure during program execution, for example, memory allocation errors, redundant code, etc. The syntax error problem is relatively well studied; most compilers are able to catch syntax errors, and correcting syntax errors manually is not difficult even for beginner programmers. The latter problem, however, is much more challenging due to several reasons. First, the error space is vast. For example, Error-Prone, a rule-based Java code error detector developed by google, identifies 499 bug patterns. Second, recognizing and correcting these bugs requires a higher level of understanding of the code, including identifying the relationship between objects, making connections between blocks, and matching data types. These errors could be seen in even experienced programmers and can be time consuming to correct manually. Therefore, this study will focus on automatic correction of these logic errors in code body that pass compiling stage.

At present, most work in this field used rule-based methods [JetBrains (2016); Synopsys (2016); Google (2016a); Google (2016b); Singh et al., (2013)], using static analyzers, code transformations, or control flow to identify bug patterns and make corrections. These methods are quite mature, and some are even commercialized, like Resharper. Machine learning methods, however, have been a minority and are relatively new. There is also no canonical solution; people have used methods varying from reinforcement learning to recurrent neural network.

Given the good performance and wide usage of rule-based PLC methods, there is a major drawback: these methods are often case specific. The developer had to design specific correction strategy for each bug pattern. For example, the core code body of Error-Prone contains 499 java script, each corresponds to a type of error. Therefore, rule-based PLC often requires large human labor to build. It also suffers from incompleteness and incapability of dealing with exceptions. In the long run, one could consider rule-based PLC vs. machine learning PLC as rule-based translation vs. statistical machine translation. Machine learning methods have the following advantages: first, they are self-sufficient; they teach themselves, requiring minimum amount of human development. Second, they can do self-improvement and self-prediction by grabbing data from users. Third, after sufficient training, one can expect them to perform better with coding style and fluency, like machine translations. One main obstacle that prevents machine code correction being as successful as machine translation is a general lack of data, which will be elaborated in a latter paragraph. This further leads to another drawback: insufficient training. However, machine code correction has an unlimited potential if more studies are carried out and more datasets are produced. This article aims to provide a successful example that might inspire further researches on machine code correction.

Despite good intentions of replacing hand-designed rule-based PLC method with machine-learning-based PLC method and its merits discussed above, some may express concerns about its environmental costs, as such concerns have been raised by ethical AI researchers (Hao, 2019). Although generally we do not agree that such concerns should overshadow the value of liberating human labor and pursuing potentially much better performances (as one did in machine translation), we leave such judgment to our readers. Since training a machine learning model takes mostly electricity and storage space, we provide an estimated power consumption and the detailed information of a number of parameters in our models (with chosen hyper parameters described in Section 3.6) in the Appendix: Section 2. Interested readers could refer to the information accordingly.

**FIGURE 1:**
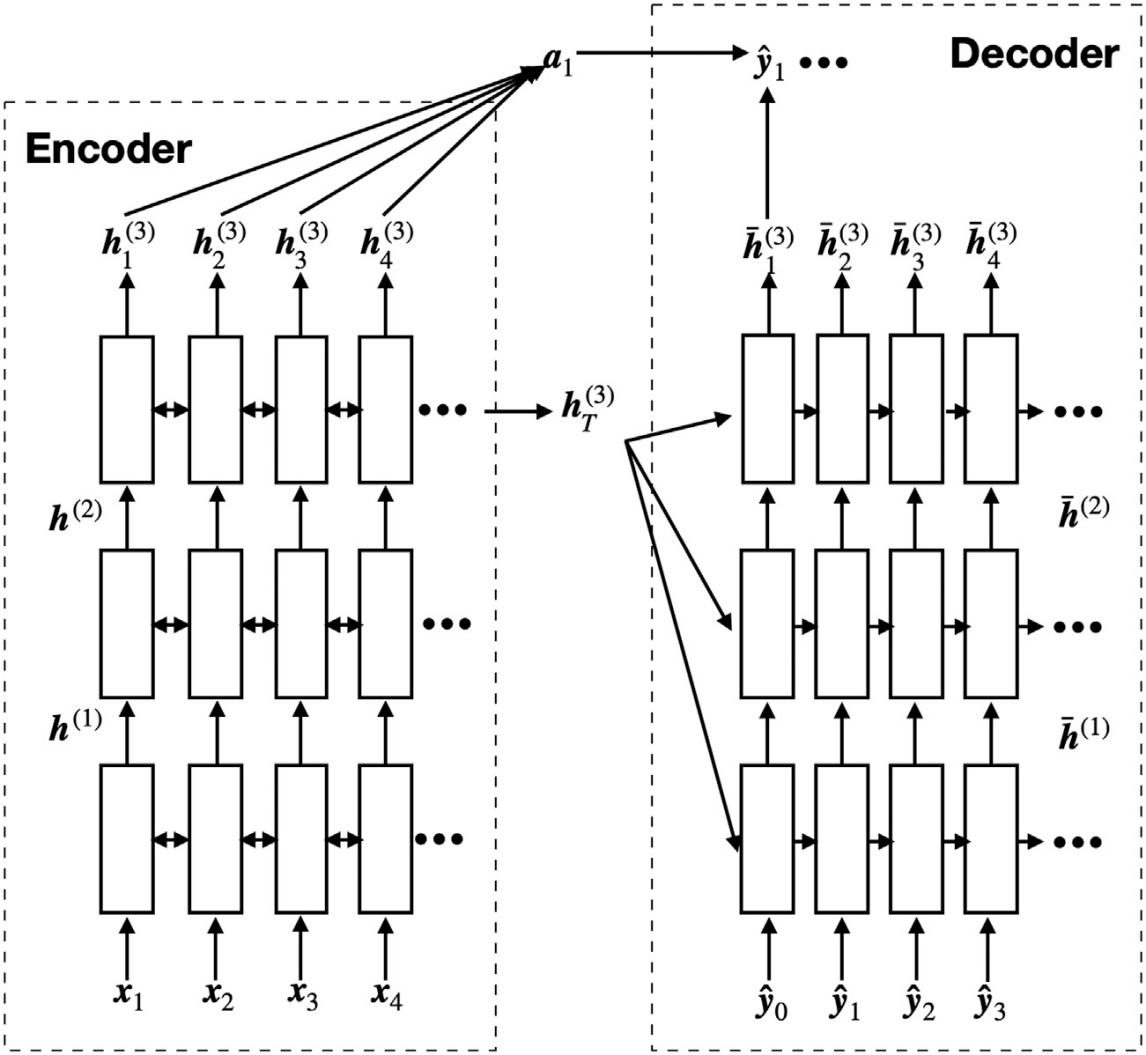
Model structure of a 3-layer seq2seq model with attention. The $i^{\text{th}}$ layer takes the output of the previous layer ($h^{\left(i-1\right)}$) as its input. ***a*** is the context vector, which can be calculated using different attention mechanisms.

The machine learning models we choose are seq2seq models. Seq2seq (abbreviation of sequence to sequence) model is a group of neural-network-based models. It usually consists of an encoder and a decoder. The encoder takes a sequence as input and produces an encoded representation of the input sequence. The decoder takes this representation and produces an output sequence. It has been proved to be very successful in neural machine translation, natural language correction, text generation, etc. An example of a seq2seq model structure is shown in Figure 1. Our results show that seq2seq models successfully repair over 70% of the code instances if the beam search size is 1 and over 90% if the beam search size is 5.

**FIGURE 2:**
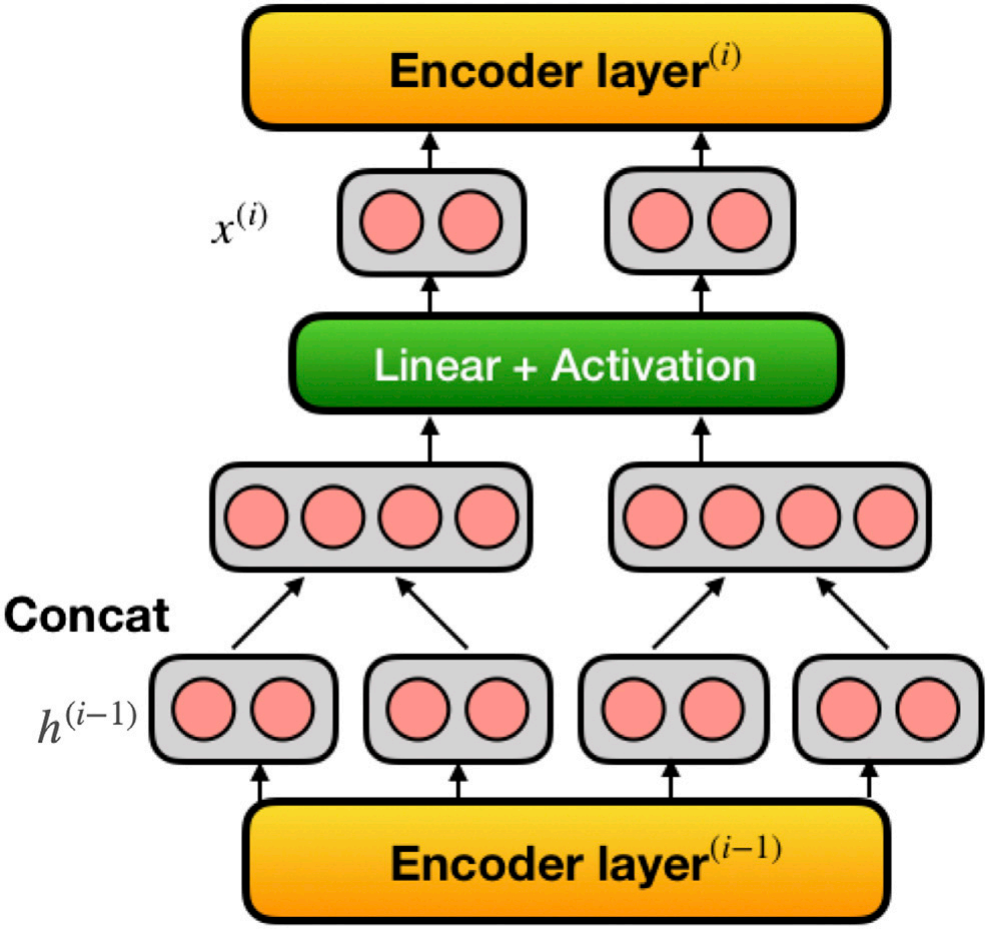
Visualization of pyramid encoder in multilayer seq2seq models. Pyramid encoder reduces length of input sequence by half in every encoding layer. $h^{\left(i-1\right)}$ denotes output of ${\left(i-1\right)}^{\text{th}}$ encoder layer and $x^{\left(i\right)}$ denotes the input of $i^{\text{th}}$ encoder layer.

Instead of just using regular seq2seq model, we introduce pyramid encoder structure to better suit the code correction task. The motivation is as follows: for NLC problems, the model works on a sentence level and the average length of a sentence lies around dozens of words. However, for PLC problems, the model works on the whole code instance. The average length of code instances in PLC is usually hundreds of syntax words, which results in enormous computational cost and memory requirement, especially combined with attention mechanisms. Pyramid structure aims to reduce these costs by contracting the data flow and discarding redundant information. Figure 2 shows a visual representation of the pyramid encoder; it can be implemented to most of the multilayer seq2seq learning models. In our model comparison set, pyramid encoder increases networks’ computational efficiency by 50%–100% and memory efficiency by up to 600%, while having similar ability of reparation.

On the other hand, due to the privacy policies, most of the publicly available datasets are not collected from realistic program errors and fixes but rather are generated by artificial tools. The ones that are collected realistically are usually very small. To handle this issue, we also applied transfer learning to inherit the knowledge learned from previous datasets to boost the network’s performance on smaller and noisier datasets. Details of our project are available on GitHub^1^.

## 2 Related Work

Rule-based methods that work on PLC have a long history and are thus more mature. One of them is proposed by Singh et al., (2013), which is a rule-directed translation strategy synthesizing a correct program from a sketch. Their model is able to provide feedback for introductory programming problems and has achieved a correction rate of 64% on incorrect submissions. Some of these methods are quite mature. For instance, Google developed Error-Prone (Google, 2016a) and clang-tidy (Google, 2016b) as rule-based tools to help in identifying and correcting potential mistakes for programmers. Some of them are even commercialized, like Resharper (JetBrains, 2016), developed by Synopsys (2016). As a paid feature of Visual Studio, Resharper provides code analysis, refactoring, and code processing (including code generation and quick fixes for errors) as extra features to programmers.

In 2016, Pu et al.’s (2016) study became one of the first attempts to use machine learning method in PLC tasks. They used a Long Short Term Memory (LSTM) model on correcting MOOCs student assignment submissions. However, their dataset was not publicly available, putting difficulties on reproducing their work. Later in 2017, Gupta et al., (2017) proposed a seq2seq model for fixing student submissions (Deepfix), which is also a private dataset. In a later work, they (Gupta et al., 2018) used reinforcement learning based on the input code and the error messages returned by the compiler for the same task, on the same dataset. Our work, also based on seq2seq models, was carried out on a public dataset that contains more error categories.

The pyramid encoder played an important role in our research. It originated from Xie et al., (2016). We proposed its general form for all seq2seq models and thoroughly studied its performance in reduction of computational resources. We aimed to overcome difficulty brought by the extended length of code instances, compared to natural language sentences. These aspects of pyramid structure were not studied in Xie’s work. We did the comparison of pyramid encoder and regular encoder under different attention mechanisms, showing that pyramid encoder could drastically reduce memory and computational cost in most setups that we considered.

## 3 Model

### 3.1 Overview

Given a code instance, we wish to identify and correct potential flaw in it, which might lead to a failure in execution after successful compilation. Each bad code instance contains exactly one flaw.

Formaly speaking, given an input code instance *x*, we wish to map it to an output code instance *y* and we seek to model P(y|x). A code is “repaired” if the flaw that *x* contains is fixed in the output *y*. The “repair rate” is defined as the fraction between the number of code instances fixed and the total number of code instances that the model was applied on. We use repair rate as the evaluation metric in our experiments.

For this purpose, we applied two major families of seq2seq models: GRU and Transformer. We use learnable embedding layers, which allows the model to recognize the relationship between different words in the vocabulary. For the encoder, we applied pyramid encoder, where a pyramid module is added in between layers of regular multilayer encoders. For the purpose of testing generality of pyramid encoder, we combined it with different attention mechanisms.

### 3.2 Word-Level Reasoning

In language correction, character-level reasoning is a more commonly applied method, Xie et al., (2016). However, in code correction, we apply word-level models. A “word” here is defined as a code syntax (e.g., “void”, “{“, space, “ = “, “int”, newline, etc.) or a custom variable name. The reason is that the basic building blocks of a code instance are related to the syntax. In the field of programming language processing, out-of-vocabulary (OOV) is less a problem than in natural language due to a fixed syntax pool.

In order to prevent the model suffering from vast variation of variable names, we performed a certain degree of variable renaming. We focused on renaming function names in our dataset while keeping other variables unchanged. This method reduced vocabulary size to ∼1,000 and was proven to be effective in improving the performance.

We include our preprocessing method to a code instance in the Appendix.

### 3.3 Pyramid Encoder

Given a multilayer seq2seq encoder, its input at $i^{\text{th}}$ layer at step *t* is $x_{t}^{\left(i\right)}$ and the output is $h_{t}^{\left(i\right)}$:

$$h_{t}^{\left(i\right)}={\text{Layer}}^{\left(i\right)}\left(x_{t}^{\left(i\right)}\right)$$ (1)

In standard seq2seq models, the output of the $i^{\text{th}}$ layer $h^{\left(i\right)}$ is directly used as input of the $i+1^{\text{th}}$ layer, $x^{\left(i+1\right)}$:

$$x_{t}^{\left(i+1\right)}=h_{t}^{\left(i\right)}$$ (2)

and the time step t=1,2,…,T, the layer number i=1,2,…,N. Note that $x_{t}^{\left(0\right)}$ is the embedded representation of the input instance.

For pyramid encoder, we introduce a pyramid module in between $h^{\left(i\right)}$ and $x^{\left(i+1\right)}$ as Eq. 3 follows:

$$x_{t\prime }^{\left(i+1\right)}=\text{tanh}\left(W_{\text{pyr}}\left(h_{2t}^{\left(i\right)},h_{2t+1}^{\left(i\right)}\right)+b_{pyr}\right)$$ (3)

This module reduced the length of the input $x^{\left(i\right)}$ by half each time it is applied. The length of final output of the encoder is $T/2^{N-1}$. One could also take a bigger window such as 3, 4, 5… depending on their needs. The hope is that pyramid structure will extract the important information and reduce the redundant information of each of the neighboring hidden state, therefore reducing the training cost while keeping the accuracy of the correction. This is conceptually similar to a convolution, but without using filters.

For our GRU models, we used multilayer bidirectional GRU and we implemented pyramid encoder as described first in Xie et al., (2016):

$$f_{t}^{\left(i\right)}=\text{GRU}\left(f_{t-1}^{\left(i\right)},x_{t}^{\left(i\right)}\right)$$ (4)

$$b_{t}^{\left(i\right)}=\text{GRU}\left(b_{t+1}^{\left(i\right)},x_{t}^{\left(i\right)}\right)$$ (5)

$$h_{t}^{\left(i\right)}=f_{t}^{\left(i\right)}+b_{t}^{\left(i\right)}$$ (6)

$$x_{t\prime }^{\left(i+1\right)}=\text{tanh}\left(W_{\text{pyr}}\left(h_{2t}^{\left(i\right)},h_{2t+1}^{\left(i\right)}\right)+b_{pyr}\right)$$ (7)

where $x_{t\prime }^{\left(i+1\right)}$ denotes the input to next layer, $f_{t}^{\left(i\right)}$ and $b_{t}^{\left(i\right)}$ denote output from a forward and a backward GRU, respectively. GRU (Gated Recurrent Unit) is a RNN (Recurrent Neural Network) type model that includes a gating mechanism in the following equations (Cho et al., 2014):

$$r_{t}=\sigma (W_{ir}x_{t}+b_{ir}+W_{hr}{\tilde{h}}_{t-1}+b_{hr}$$ (8)

$$z_{t}=\sigma (W_{iz}x_{t}+b_{iz}+W_{hz}{\tilde{h}}_{t-1}+b_{hz}$$ (9)

$$n_{t}=\text{tanh}\left(W_{in}x_{t}+b_{in}+r_{t}\text{*}W_{hn}{h˜}_{t-1}+b_{hn}\right)$$ (10)

$${\tilde{h}}_{t}=\left(1-z_{t}\right)\text{*}n_{t}+z_{t}\text{*}{h˜}_{t-1}$$ (11)

where ${\tilde{h}}_{t}$ is the hidden state at step *t*, which is denoted by $f_{t}$ in Eq. 4 and $b_{t}$ in Eq. 5. $r_{t}$, $z_{t}$, and $n_{t}$ are the reset, update, and new gates, respectively. *σ* is the sigmoid function.

**FIGURE 3:**
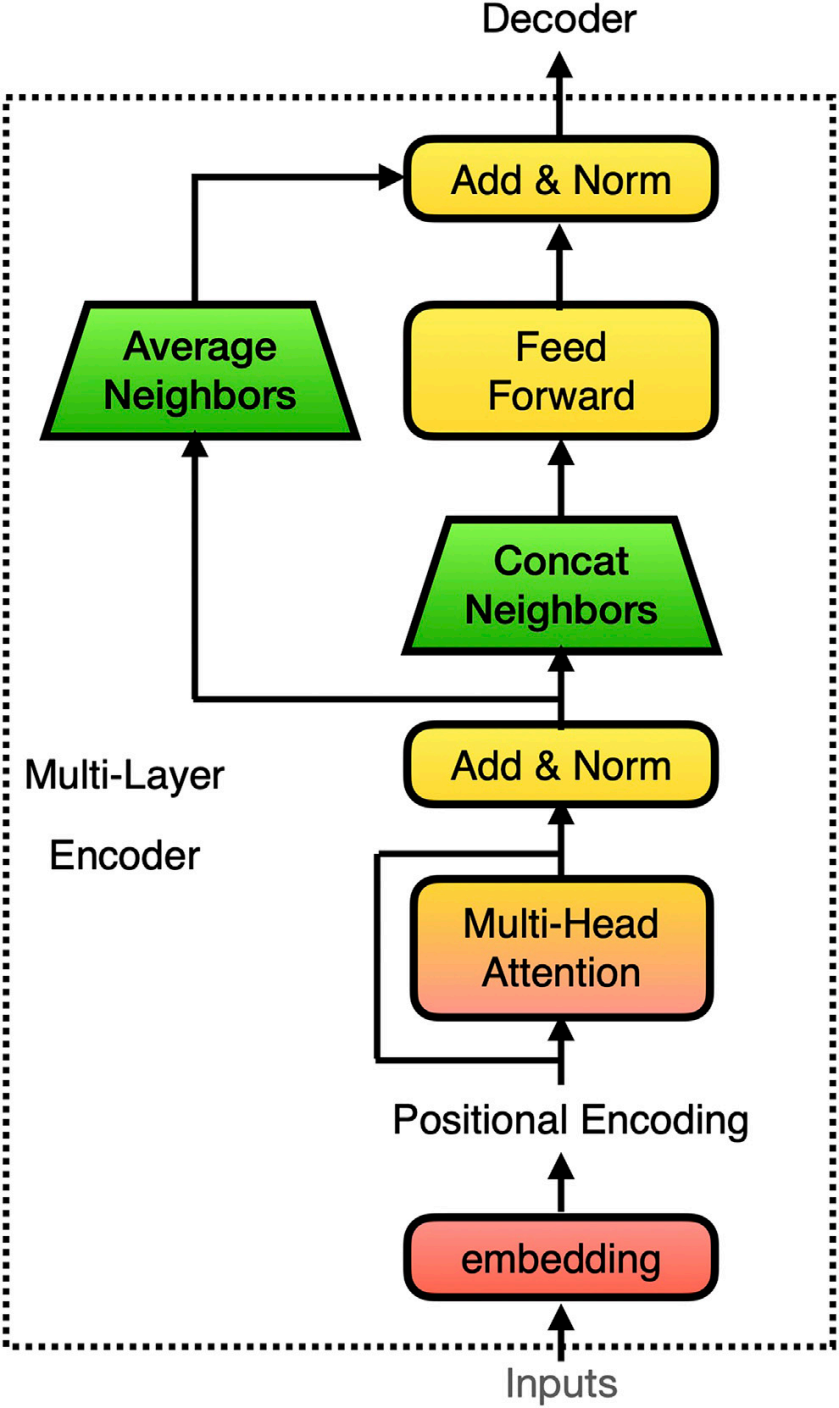
The implementation of pyramid structure in Transformer’s encoder.

Transformer is a novel family of seq2seq model that works very differently than RNN type models. In the original Transformer (see Figure 3), a Feed Forward layer directly takes in the output from the Multihead attention layer $c_{\text{att}}$, accompanied by a residual connection, shown in

$$c_{\text{att}}^{\left(i\right)}=\text{MultiHeadAtt}\left(x^{\left(i\right)}\right)+x^{\left(i\right)}$$ (12)

$$x^{\left(i+1\right)}=c_{\text{att}}^{\left(i\right)}+\text{FeedForward}\left(c_{\text{att}}^{\left(i\right)}\right)$$ (13)

In our model, we concatenated the neighboring elements in $c_{\text{att}}$ before we feed it into the Feed Forward. As a result, the dimension of the first Linear layer in the Feed Forward layer has to change from $\left[d_{\text{model}}\times d_{\text{ff}}\right]$ to $\left[2d_{\text{model}}\times d_{\text{ff}}\right]$. Here we use the same notation as in Vaswani et al., (2017), where $d_{\text{model}}$ is the size of input, output, and attention vectors and $d_{\text{ff}}$ is the number of neurons in the Feed Forward layer. The residual connection also has to be changed accordingly; we tried two different approaches, simply averaging the neighboring element (Eq. 14) or concatenating the neighboring element and passing it through another affine transformation to recover its dimensions (Eq. 15). For simplicity, we denote the former method with subscript “ave” and the latter with subscript “aff”.

$$x_{t^{\prime },\text{ave}}^{\left(i+1\right)}=\frac{\left(c_{\text{att},2t}^{\left(i\right)}+c_{\text{att},2t+1}^{\left(i\right)}\right)}{2}+\text{FeedForward}\left[\left(c_{\text{att},2t}^{\left(i\right)},c_{\text{att},2t+1}^{\left(i\right)}\right)\right]$$ (14)

$$x_{t^{\prime },\text{aff}}^{\left(i+1\right)}=\text{tanh}\left[W_{\text{aff}}\left(c_{\text{att},2t}^{\left(i\right)},c_{\text{att},2t+1}^{\left(i\right)}\right)+b_{\text{aff}}\right]+\text{FeedForward}\left[\left(c_{\text{att},2t}^{\left(i\right)},c_{\text{att},2t+1}^{\left(i\right)}\right)\right]$$ (15)

In our experiments, both methods show close performance. Therefore when showing the results, unless otherwise specified, we use the results of “ave” version.

### 3.4 Decoder and Attention Mechanisms

For our GRU models, we compared a regular multilayer unidirectional GRU:

$${\bar{h}}_{t}^{\left(i\right)}=\text{GRU}\left({\bar{h}}_{t-1}^{\left(i\right)},{\bar{h}}_{t}^{\left(i-1\right)}\right)$$ (16)

In our experiment, we did a comparison study on Bahdanau attention (Eq. 17) and different Luong attentions. Bahdanau attention is described in following set of equations.

$$u_{tk}={\left(W_{1}{\bar{h}}_{t}^{\left(M\right)}+b_{1}\right)}^{⊤}\left(W_{2}h_{k}^{\left(N\right)}+b_{2}\right)$$ (17)

$$\alpha_{tk}=\frac{u_{tk}}{\sum_{j}u_{tj}}$$ (18)

$$a_{t}=\sum_{j}\alpha_{tj}h_{j}^{\left(N\right)}$$ (19)

Here, *u* is the alignment score, *h* and $\bar{h}$ denote the hidden state in encoder and decoder, respectively. *M*, *N* are the number of layers in decoder and encoder, respectively. $a_{t}$ is the context vector, which will be concatenated with the decoder hidden state of last layer for predicting the next word $\hat{y_{t}}$.

Luong’s global attentions are generalizations to Bahdanau attention, but using different alignment score calculation methods. For simplicity, we omit the superscript (M) and (N).

$$u_{tk}=\{\begin{array}{ll} {\bar{h}}_{t}^{⊤}h_{k} & \text{dot}\\ {\bar{h}}_{t}^{⊤}W_{a}h_{k} & \text{general}\\ v_{a}^{⊤}\text{tanh}\left(W_{a}\left[{\bar{h}}_{t},h_{k}\right]\right) & \text{concat} \end{array}$$ (20)

We also tried one example of Luong’s local attention, which is done by imposing a Gaussian on Eq. 19 at a desired attention center $p_{t}$:

$$a_{t}=\underset{j}{\sum }\left(\alpha_{tj}h_{j}\right)\text{exp}\left(-\frac{{\left(j-p_{t}\right)}^{2}}{2\sigma^{2}}\right)$$ (21)

$$p_{t}=S\cdot \text{sigmoid}\left(W_{p}{\bar{h}}_{t}\right)$$ (22)

where *S* denotes the total length of the hidden state from the last encoding layer and *σ* is a parameter chosen manually.

### 3.5 Beam Search

We use beam search in test and validation where text generation is involved. For each time step, we rank candidates based on their total negative logarithmic probability to current decoding time step $t_{\text{dec}}$:

$$\text{score}=-\overset{t_{\text{dec}}}{\underset{t}{\sum }}\text{log}\left(P\left(\hat{y}\right)\right)$$ (23)

The search stops when there are five completed candidates.

### 3.6 Model Parameters

In all our experiments, we used a learnable embedding layer which embeds each “word” into a vector of length 400.

In our GRU models, we used a 3-layer bidirectional encoder; the size of the hidden states are 400 in all three layers. We used a 3-layer unidirectional decoder; the size of the hidden states are also 400.

In our Transformer models, following the original study, we used $d_{\text{model}}=512$ and $d_{ff}=2048$. We used 3-layer encoder and 3-layer decoder.

We did a coarse parameter space search to find these parameters chosen to be roughly optimal. But we did not fine-tune these parameters, because (1) we show that the overall performance of seq2seq model on PLC problem is satisfying and (2) we are more concerned about comparison between different attention mechanisms and between pyramid encoder and regular encoder.

## 4 Datasets

We perform our experiments mainly on the Juliet Test Suite for C/C++ (v1.2) (created by NSA Center for Assured Software (2013)). This dataset contains 61,387 test cases, each test case contains one flawed code instance and one to several repaired code instance. These test cases contain more than 100 Common Weakness Enumerations (CWEs); each of them contains hundreds of example code instances. We note that the instances contain significant amount of dead code. To make the code more realistic, we remove the dead code. We also found that many of the code instances contain “if conditions”, that, in the flawed code instance, executes one branch, while, in the repaired instance, executes the other. These instances are unrealistic; therefore, we removed them. We also performed function renaming. After the preprocessing, we obtained 31,082 pairs of good-bad code instances.

To test model’s generality, for some of the models, we also tested their performance on Juliet Test Suite for Java (v1.3) (released by NSA Center for Assured Software (2018)). After similar preprocessing described above, we obtain 23,015 pairs of instances.

We did 4-fold cross-validation in all of our experiments to achieve statistically accurate results. An estimation of time and power consumption when running our experiments is provided in the Supplementary Material in a table, along with hardware requirements.

## 5 Results

### 5.1 Repair Rate

We train our models on a GeForce GTX 1080 Ti graphic card. The metric we use for evaluation is the repair rate, which is the fraction of instances that are repaired after the model’s edit. Since we performed beam search with beam width 5, each time a correction is being performed, we generate five correction candidates. Here we have two metrics in measuring the performance: one-candidate repair rate and five-candidate repair rate. The former corresponds to the scenario of code autocorrection, where there is no human judgment involved. The latter corresponds to correction suggesting, where the machine will identify an error and provide suggestions for the programmer for further judgment. The comparisons of the repair rates for considered models and their counterparts with pyramid encoder are listed in Table 1 and Table 2. For comparison, we have attempted to test other machine-learning-based PLC tools that have been made. Gupta et al., (2018) take error messages while compiling as input, but our dataset focuses on logic flaws in programs that do not have syntax errors; therefore, this tool is not applicable. Pu et al., (2016) do not provide an open source repository, nor any documentations of their code. We have successfully trained Gupta et al., (2017) on our C/C++ dataset and included it in our work for comparison. Unfortunately, a tokenizer is required for preprocessing the data into a certain format, and they only provided that for C/C++, but not java.

**TABLE 1:** Repair rate of GRU and Transformer on Juliet Test Suite for C/C++, comparing the regular encoder and pyramid encoder. These results are averaged over a 4-fold cross-validation. We calculated the improvement of pyramid encoders compared to their nonpyramid pairs. Apparently pyramid encoder does not collaborate well with Luong’s local attention; therefore, we exclude it from future discussions. It is also not included when calculating the average improvement.

Model	1-Candidate repair rate (%)		5-Candidate repair rate (%)	
	Regular encoder	Pyramid encoder	Regular encoder	Pyramid encoder
GRU + Bahdanau Att	76.92	76.09 (−0.83)	96.19	95.55 (−0.64)
GRU + Luong Att: Dot	74.38	73.04 (−1.34)	94.27	94.59 (+0.32)
GRU + Luong Att: General	75.79	74.85 (−0.94)	94.83	94.92 (+0.09)
GRU + Luong Att: Concat	50.34	47.26 (−3.08)	86.72	86.14 (−0.58)
GRU + Luong Att: Local	65.70	49.18 (−15.52)	92.46	86.24 (−6.22)
Transformer	75.48	72.39 (−3.09)	97.66	96.78 (−0.88)
Average improvement (%)	−1.95		−0.34	

**TABLE 2:** Repair rate of GRU and Transformer on Juliet Test Suite for Java, comparing the regular encoder and pyramid encoder. We did not include result from DeepFix, because the provided data tokenizer only support C/C++.

Model	1-Candidate repair rate (%)		5-Candidate repair rate (%)	
	Regular encoder	Pyramid encoder	Regular encoder	Pyramid encoder
GRU + Bahdanau Att	54.65	56.21 (+1.56)	84.31	83.98 (−0.33)
GRU + Luong Att: Dot	54.30	55.66 (+1.36)	82.73	84.86 (+2.13)
GRU + Luong Att: General	53.15	52.54 (−0.61)	82.81	82.83 (+0.02)
GRU + Luong Att: Concat				
Transformer	56.68	57.35 (+0.67)	93.11	93.54 (+0.43)
Average improvement (%)	+0.74		+0.75	

From these results we see that pyramid encoder has close performance to regular encoder in most of the models we applied to, except for Luong’s local attention. The reason is that the encoder output in pyramid encoder is very “coarse-grained”; each output position now represents information from $2^{\left(N-1\right)}$ words. This results in two drawbacks specifically to local attention: one, a much more “blurry” attention center and two, a much broader attention window. As a result, the attention is much less targeted, which damages the performance. Therefore, in the rest of the article, we will exclude this attention mechanism from our discussion.

### 5.2 Converging Speed

Since pyramid encoder reduces the sequence lengths in higher layers, one can expect a smaller training cost per batch in both GRU and Transformer models. To quantify this effect, for each of the regular encoder-pyramid encoder model pairs in Table 1, we set the same batch size and compare the average training speed in words per second, as shown in Table 3. Here the batch size is chosen so that it optimizes the training speed on the given GPU for each model. In the model, we also included number of epochs for the model to converge.

**TABLE 3:** Training speed of GRU and Transformer on Juliet Test Suite for C/C++.

Model	Batch size	Training speed (words/s)		Converge epoch	
		Regular	Pyramid	Regular	Pyramid
GRU + Bahdanau Att	8	754	1,185 (+57%)	18	18
GRU + Luong Att: General	16	441	853 (+108%)	23	27
GRU + Luong Att: Dot	128	4,646	10,408 (+124%)	36	34
GRU + Luong Att: Concat	6	1,418	2,344 (+65%)	23	29
Transformer	8	1,086	2,181 (+101%)	33	34

**FIGURE 4:**
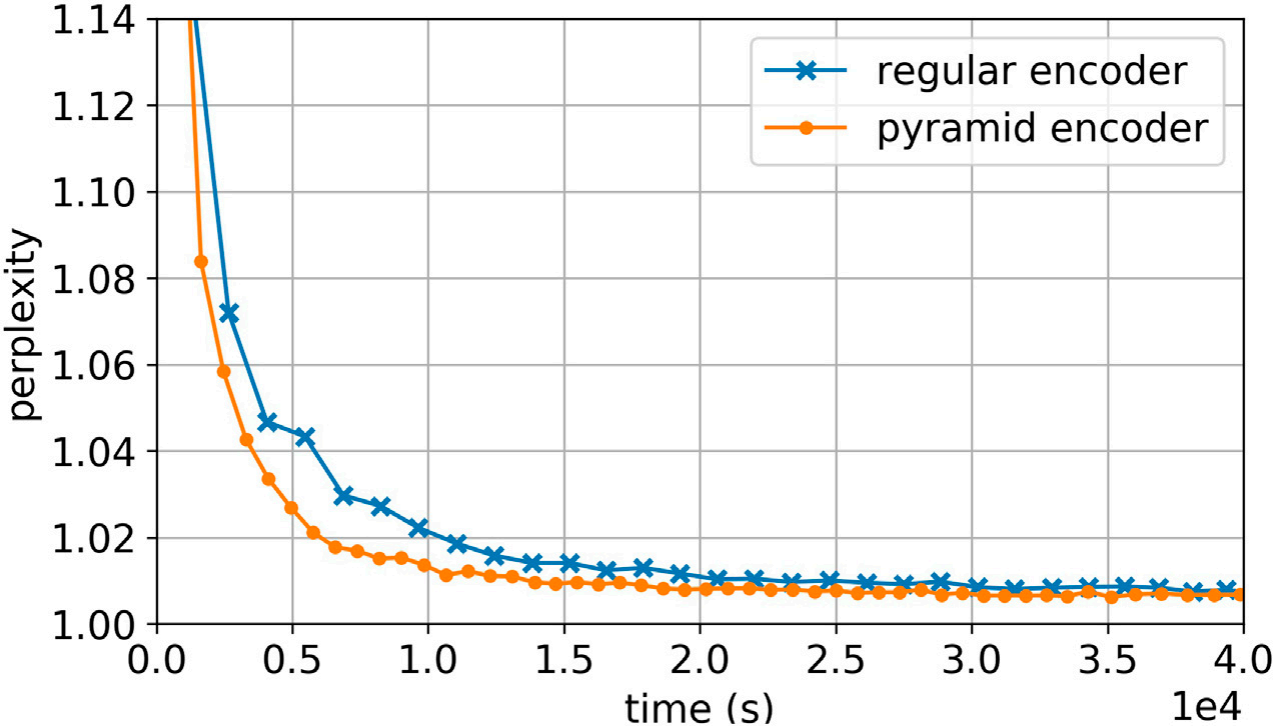
Learning curve of GRU with Luong’s general attention, comparing regular encoder to pyramid encoder. Pyramid encoder model shows fast converging speed.

Apparently it takes similar number of epochs to converge for the same type of model with pyramid encoder and regular encoder. However, pyramid encoders largely increase the training speed, between 50 and 130%. Therefore it could easily shorten the training time by two to four folds while the same performance is achieved. As an example, Figure 4 shows the learning curve for GRU model with general Luong’s attention, comparing the regular encoder and pyramid encoder.

### 5.3 Memory Cost

The last thing we compared is the memory cost of the pyramid encoder and the regular encoder. This measure is crucial in some scenarios, where your input instances are very long; therefore, the memory of GPU is only capable of holding a very small batch. In code correction, this is often the case.

The metric we use for comparison is memory cost per instance, *k*, which is defined as

$$k=\frac{\text{ΔMemory usage}}{\text{ΔBatch size}}$$ (24)

**FIGURE 5:**
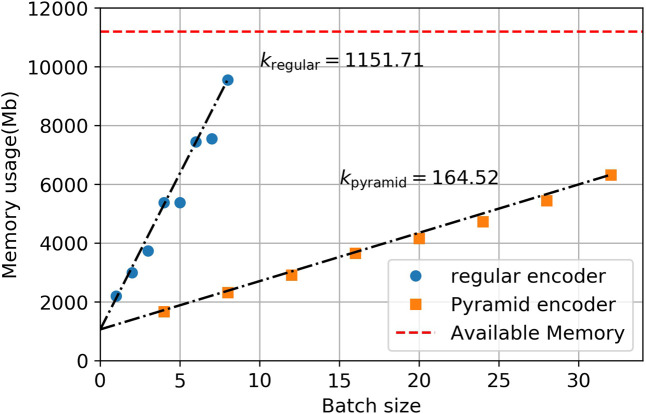
Memory cost per instance for GRU models with Bahdanau attention, *k* is calculated by finding the slope of the linear fit (black dashed line). The red dashed line represents the maximum memory of a GeForce GTX 1080 Ti graphic card.

Figure 5 shows the calculation process of *k*. Define ℰ=1/k as memory efficiency. We calculated the *k* and ℰ value for each of the models we applied, shown in Table 4. We also included the number of parameters in each model, from which we see that each pair of models has roughly the same model size.

**TABLE 4:** Memory cost for considered models, comparing regular encoder and pyramid encoder: pyramid encoder greatly increased the memory efficiency.

Model	k (Mb/instance)		$ℰ\left(10^{-3}\right)$		Parameters (10^7^)	
	Regular	Pyramid	Regular	Pyramid	Regular	Pyramid
GRU + Bahdanau Att	1,151.71	164.52	0.86	6.08 (+600%)	1.24	1.11
GRU + Luong Att: General	830.71	165.03	1.20	6.05 (+403%)	1.22	1.10
GRU + Luong Att: Dot	65.91	52.42	15.17	19.08 (+26%)	1.20	1.08
GRU + Luong Att: Concat	1,381.6	431.87	0.72	2.31 (+220%)	1.24	1.11
Transformer	414.67	263.33	0.24	0.38 (+57%)	2.35	2.82

The pyramid encoder could increase the memory efficiency by 20%–600% depending on the attention mechanisms used, while only increase the memory occupied by the model itself by around 10%. One should note that the memory efficiency directly affects the maximum batch size one is able to use on a single GPU, and therefore affects the utility of the GPU. For example, for regular GRU with Bahdanau Attention, the memory of a GeForce GTX 1080 Ti graphic card can only support a batch size of 8, which does not fully utilize the GPU. With pyramid encoder, it can support up to 60 instances each batch. In practice, this will drastically reduce the training time by increasing the GPU utility, together with the smaller computational cost of pyramid encoder as addressed in previous section.

## 6 Discussion

### 6.1 Length Analyses

**FIGURE 6:**
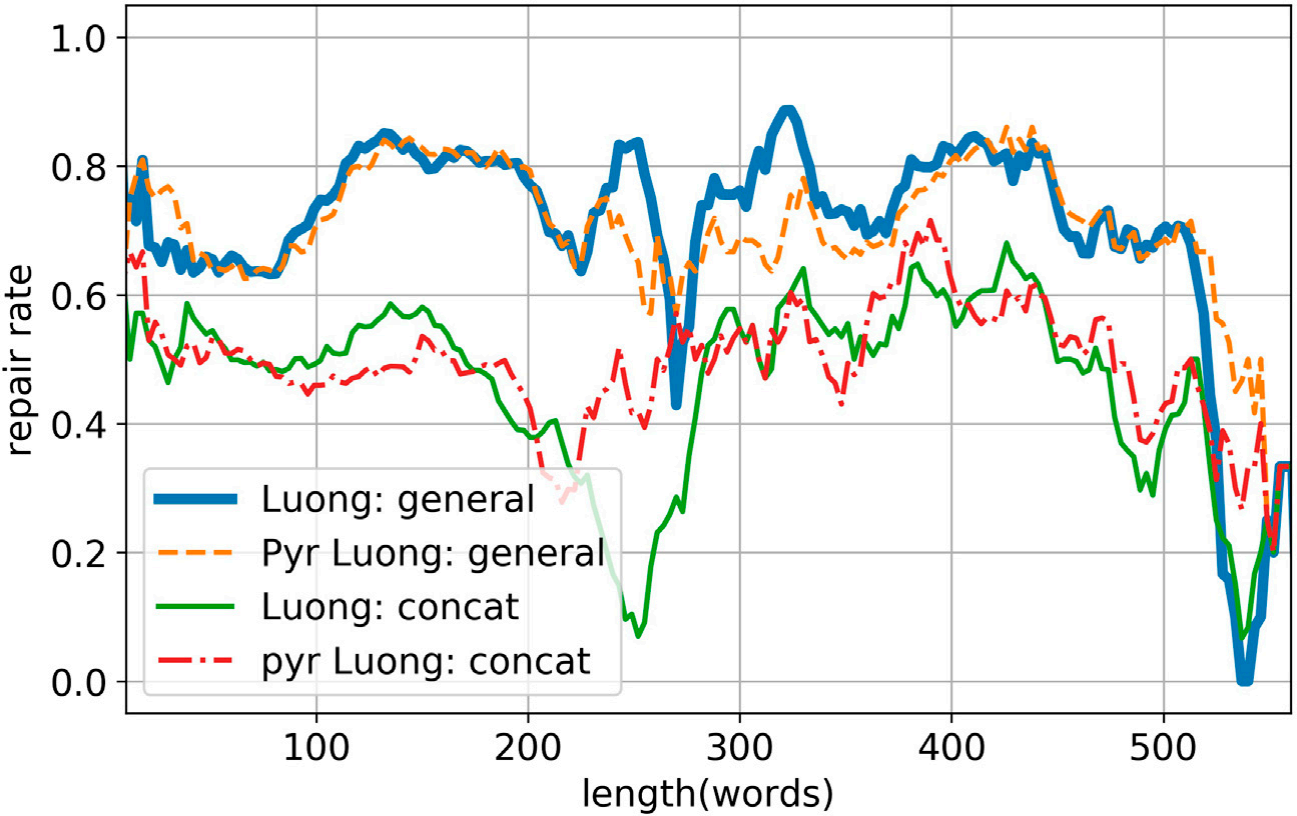
Length analyses of Luong’s general attention and Luong’s concat attention. The results from the rest of the models are qualitatively similar to result of Luong’s general attention and thus are omitted.

**FIGURE 7:**
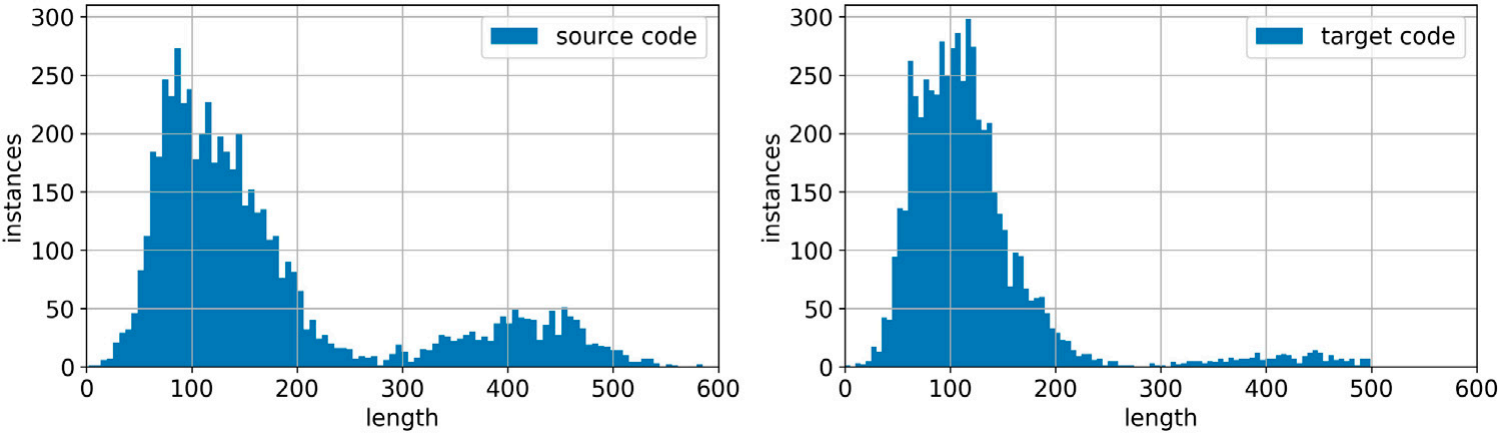
Histogram of flawed code (left) and repaired code (right) instances.

Figure 6 shows the repair rate of the models with respect to the input length. We omitted the result of Transformer, Bahdanau’s attention, and Luong’s general attention, because they are qualitatively similar to the result of Luong’s dot attention. Despite the different attention mechanisms, these seq2seq models (with pyramid encoder or regular encoder) are relatively robust to longer input lengths. The performance drops at around 250 words and above 500 words are likely resulting from the shortage of samples, which one can easily observe from Figure 7, the length histogram of source instances and target instances. The histogram also shows that the majority of code instances contains several hundred words, while natural language sentences are typically not longer than 50 words. This feature of code instances calls for a much higher computational resource requirement for PLC problems than NLC problems, which makes pyramid structure especially useful.

### 6.2 Examples of Correction

In this section we give several examples of successful corrections from our Pyramid GRU model on Juliet C/C++ Test Suite for closer examination of model and datasets. The red striked out texts denote the original faulted instance, and blue buffed texts are the reparation done by the model.

Example 1: Memory allocation match

The flawed code creates a char variable whose size does not match its concatenating destination. The model is able to correct it so that their size matches each other.

Example 2: Redundant Code

This is an example that the model deletes repeated code where a variable is freed twice.

**Figure:**
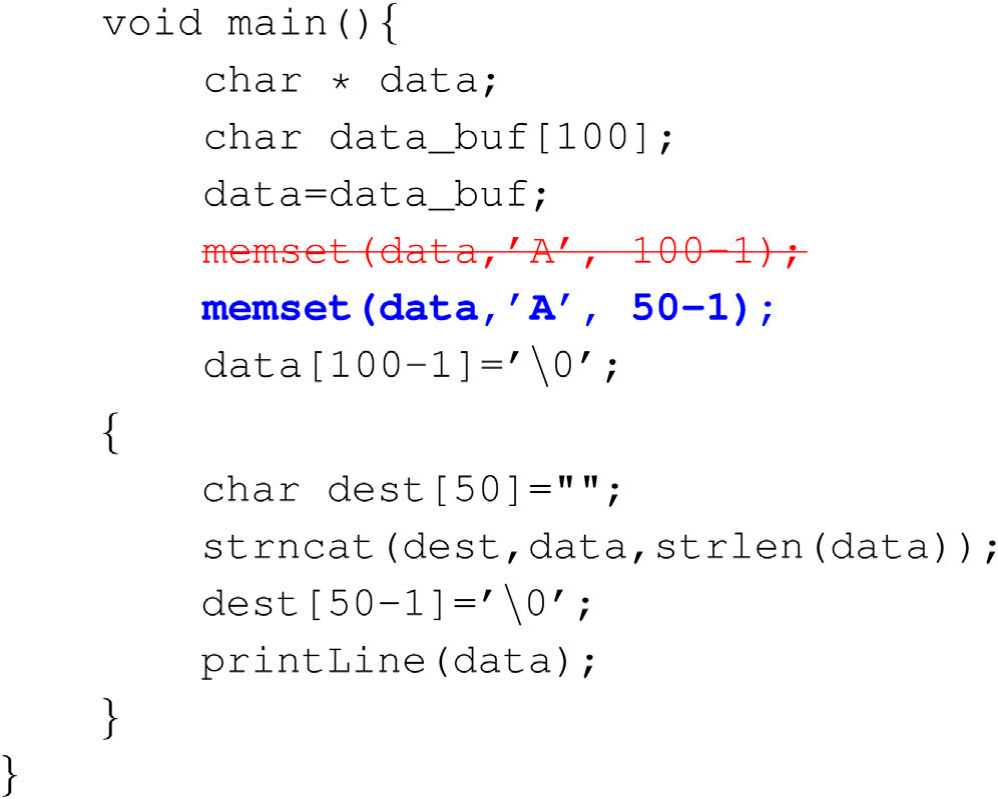

Example 3: Possible Overflow

Here we show a slightly questionable example of correction provided by the dataset. In order to prevent potential string overflow emerging from environment variable, the repair suggestion given by the Juliet Test Suite is to abort the entire part of concatenating the environment string and replace the variable with an arbitrary string “*.*”. This “correction” is easy for the model to learn; however, it has changed the original purpose of the program.

**Figure:**
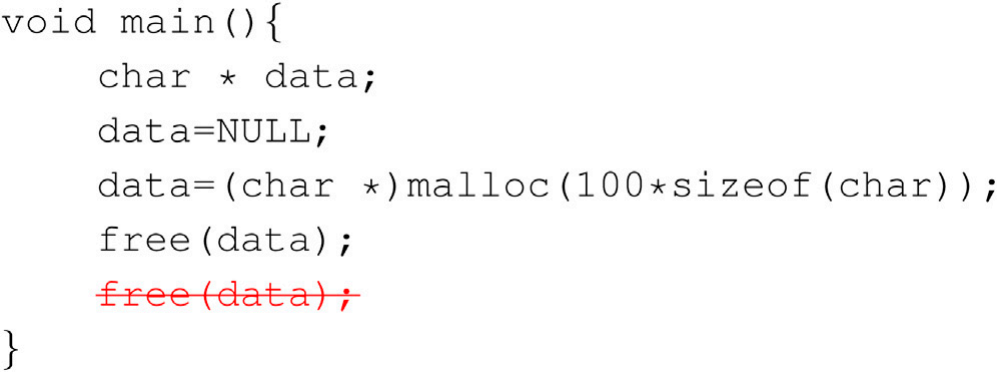

Example 4: Correction Across Functions

In this example, the models demonstrate the ability of making connections across the whole instance, between different functions. Here it prevents potential overflow in the sink function caused by a variable that was passed from the main function by adding an “if condition”.

**Figure:**
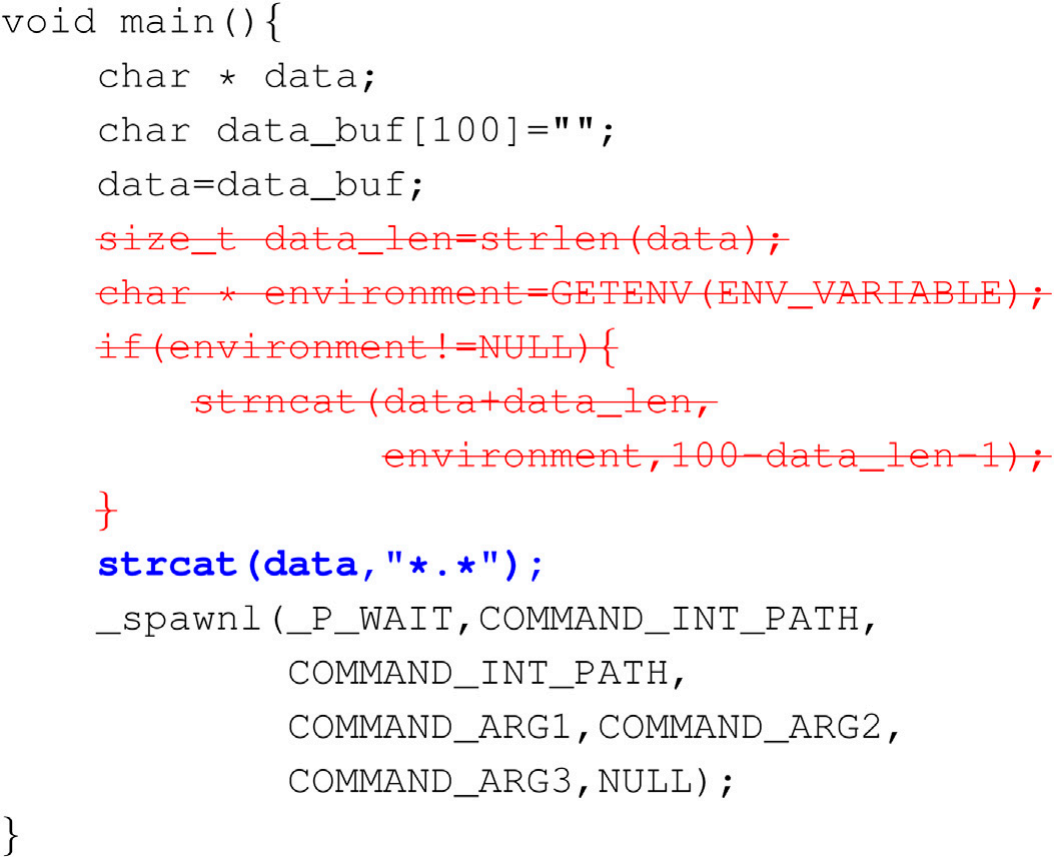


**Figure:**
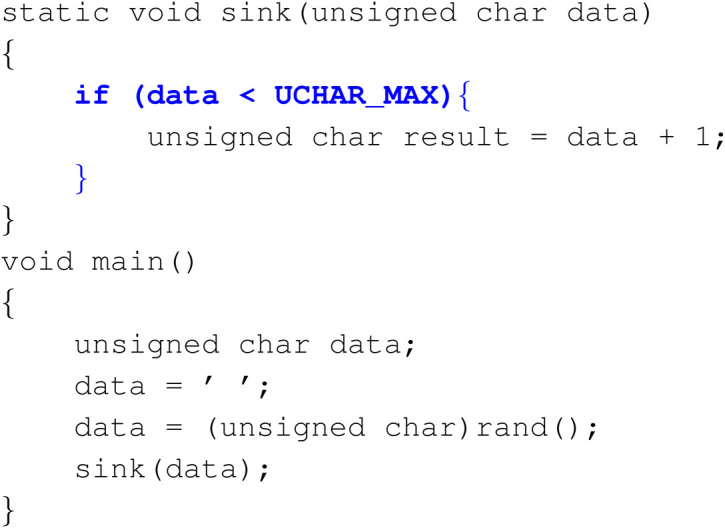


### 6.3 Generalizability to Syntax Error-Oriented Dataset

In the spirit of comparative study, we attempted to compare our method to Deepfix (Gupta et al., 2017), the only machine-learning-based PLC method that made their code and dataset open to the public, to the best of our knowledge. Unfortunately, the attempt of applying Deepfix onto Juliet Test Suite has failed, because Deepfix is aimed only to correct syntax errors and a compiler is used as the evaluator, marking any programs that could pass the compiling stage as “correct”. This apparently contradicts the spirit of “identify logic errors from syntactically correct programs”.

The difficulty that we are facing here comes from a more general problem in the field of Machine Learning PLC; the field is still disorganized and works in the field are uncorrelated. Each group might be using their own dataset and design their systems to match the specific purpose of that dataset. Comparative work is difficult to conduct not only because the datasets are hard to obtain due to private policies, but also because issues raised in PLC are versatile; each model is designed and optimized to best address the problem occurring in their particular dataset.

For the above reasons, we had to back off to a weaker comparative study, using seq2seq models on the dataset from Deepfix. Deepfix uses a generated dataset, originated from students’ submission to an introductory C course in a web-based tutoring system (Das et al., 2016). For each student submission, they generate up to five syntax errors in the code instance, including replacing “}; ” with “; }”, deleting a semicolon, add an extra “}”, replacing a semicolon with a period, and replacing a comma with a semicolon. If all of the syntax errors were fixed, then consider such a program as successfully repaired.

Table 5 shows the comparison of repair rate of our seq2seq models compared to the method applied by Deepfix. We observe that pyramid encoder performs worse than regular encoder on this particular dataset. This is expected from how the dataset was generated. The generated syntax errors are extremely local in Deepfix’s dataset. The fix usually only involves changing one token or two neighboring tokens, leaving the rest of the entire code piece unchanged. Therefore, while a pyramid encoder summarizes the information from neighboring tokens, it also blurs the local information.

**TABLE 5:** Comparison of our models with Deepfix, Gupta et al., (2017) on Deepfix dataset. All results are average of 5-fold cross validation.

Model	1-Candidate repair rate (%)		5-Candidate repair rate (%)	
	Regular encoder	Pyramid encoder	Regular encoder	Pyramid encoder
Transformer	51.96	43.78	67.16	59.32
GRU + Luong Att: General	51.86	34.80	66.33	48.44
GRU + Luong Att: Dot	58.63	41.09	72.31	54.47
GRU + Bahdanau Att	27.47	15.21	36.19	22.59
Deepfix	56			

We also observed that, in Luong’s attention, dot has the best performance in this dataset and Bahdanau’s attention performs the worst. After observing the dataset carefully, we came up with the following hypothesis: in this dataset, the network is only required to simply **copy** the original token most part of the instance and locally fix one or two tokens. This means that in the majority of times, for each decoder hidden state ${\bar{h}}_{t}$, the normalized attention score $\text{score}\left({\bar{h}}_{t},h_{t'}\right)$ needs to be close to one where t=t' and close to 0 everywhere else. In Luong’s attention, a dot, which simply do an inner product of hidden states, could do the job easier, because latent vectors are mostly orthogonal to each other in the latent space due to high dimensionality. On the other hand, Bahdanau attention, which does an affine transformation to every hidden state $h_{t}$, may overcomplicate the problem and fail to capture the correct attention.

### 6.4 Alternative Method for Small Datasets: Transfer Learning

One main difficulty that researchers often come across when attempting to apply machine learning methods to PLC problems is the availability of suitable datasets. Although there are many datasets and shared tasks available on Software Assurance Reference Dataset (2006), most of them include less than 1,000 examples. This makes neural-network-based methods nearly impossible. To tackle this problem, we take the idea of transfer learning from Pan and Yang (2009).

Our idea is to take the encoder part of the model that was trained on Juliet Test Suite and attach it to a untrained decoder, which was designed for the specific problem. We aim to take the advantage that codes written in the same coding language share the same syntax library and same construction rules.

Since many datasets available only provide the faulted code and their corresponding fault categories, here we give an example of fault classification using transfer learning, applying the model pretrained on Juliet Test Suite for C/C++ on ITC benchmark (Charles (2015)).

#### 6.4.1 Model Structure

Given a faulted code instance, our goal is to train a classification model that predicts the type of error of the faulted code from a given list of error categories.

We keep the encoder part of the pretrained model and use it directly as the encoder in the classification problem. The exception is the embedding layer, because the vocabulary in the new dataset will contain new variable names that did not occur in pretrained embedding, although the syntax will be the same. In practice, we manually expanded the embedding layer to accommodate the new “words” but keep the embeddings of the old “words” unchanged. In order to add variation from the original model, we also reinitialized the weights in the last encoding layer.

For the decoder, instead of generating a sequence, we take the output of the first time step of the reinitiated decoder and pass it to a linear layer that projects it to an $n_{\text{class}}$ dimensional vector. $n_{\text{class}}$ is the number of error classes. Model was trained to minimize cross-entropy loss with an ADAM optimizer.

#### 6.4.2 Results

We extracted 566 C/C++ code instances from the ITC bench mark. These instances are organized into 44 error categories, with the largest category containing around 30 instance and the smallest only containing two instances. Then the instances are divided into a training set of 485 instances, a validation set of 42 instances, and test set of 39 instances. For comparison, we also tried Pyramid GRU and Pyramid Transformer with the same model structure but no prior knowledge from Juliet Test Suites. The result is shown in Table 6.

**TABLE 6:** Comparison of the result of transfer learning on error type classification task. The models without transfer learning demonstrate no predicting power and no improvement during course of training.

Model	Accuracy (%)
Transfer learning: PyrGRU	60.5
Transfer learning: PyrTFM	69.1
Fresh pyramid GRU	16.7
Fresh pyramid transformer	7.1

For the fresh GRU and Transformer models, we observed that the models have no predicting power as it produces constant prediction over all inputs. There is even no sufficient gradient on the loss landscape as the loss did not reduce during the training. Transfer learning, on the other hand, demonstrates a fair power of prediction, correctly classifying over 60% of instances, despite that ITC benchmark is written in very different style than Juliet Test Suites and that the dataset is 50 times smaller.

This result shows that one is able to use neural-network-based methods in code correction problems despite the shortage of data, which is a common problem in this field.

## 7 Conclusion

In our work, we show that seq2seq models, successful in natural language correction, are also applicable in programming language correction. Our results shows seq2seq models can be well applied in providing suggestions to potential errors and have a decent correct rate (above 70% in C/C++ dataset and above 50% in Java dataset) in code auto-correction. Although these results are only limited in Juliet Test Suites, we expect that, given sufficient training data, seq2seq models can also perform well when applied on other PLC problems.

Based on the commonly used encoder-decoder structure, we introduce a general pyramid encoder in seq2seq models. Our results demonstrates that this structure significantly reduces the memory cost and computational cost. This is helpful because PLC are generally more computationally expensive than NLC, due to its longer average instance length.

The publicly available datasets in PLC are mostly small and noisy. Most datasets we found contain close to or less than 1,000 code instances. This is far less than enough for training seq2seq and many other machine learning models. Our results on transfer learning pointed out a way of processing these small dataset using the pretrained model as an encoder, which boosts the performance by a large amount.

In future, we will further investigate the influence of different architectures in neural networks, for instance, parallel encoders/decoders, Tree2Tree models, etc. On the other hand, instead of code correction, we will modify and examine our model’s performance on other tasks such as program generation and code optimizing. We will also examine the potential difference between artificial datasets and realistic datasets.

## Data Availability

Publicly available datasets were analyzed in this study. This data can be found here: https://samate.nist.gov/SARD/around.php#juliet_documents https://samate.nist.gov/SARD/view.php?tsID=104.

## Footnotes

1 See https://github.com/b19e93n/PLC-Pyramid.
