# Supplementary material for: Application of Seq2Seq Models on Code Correction
Source: Front Artif Intell. 2021 Mar 19;4:590215. doi: 10.3389/frai.2021.590215 (PMC8017285; doi:10.3389/frai.2021.590215)
Supplement: Supplementary file 1 [file datasheet1.pdf]

Appendix

1 PREPROCESSING OF JULIET TEST SUITE

1.1 Overview

This manuscript will illustrating our preprocessing method of the Juliet Test Suite and the computing overhead estimation and also provides a rough estimation of different methods' computational overhead.

In general, the preprocessing involves following steps:

- Identify and extract function from code files
- Delete comments
- Function name replacement
- Delete unnecessary white spaces and newlines
- Parse string into list of words

In following parts we will give an example to illustrate each step in more detail. We only present one example for Juliet C/C++ Test Suite, the Java Test Suite will be preprocessed in the similar fashion.

1.2 An Example Code Instance of Juliet Test Suites for C/C++

Following is a simple example from case *CWE390_Error_Without_Action__calloc_01.c*, after we have extract the function part of the code. If there were multiple `.c` files in one case, we concatenate their extracted functions to make it as one instance.

```
void CWE390_Error_Without_Action__calloc_01_bad()
{
    {
        char * data = (char *)calloc(100, sizeof(char));
        /* FLAW: Check to see if calloc() failed, but do nothing about it */
        if (data == NULL)
        {
            /* do nothing */
        }
        strcpy(data, "Body");
        printLine(data);
        if (data != NULL)
        {
            free(data);
        }
    }
}
```

First we remove all the comment, then replace the function name with `main`. We replace all function names as `main` to avoid the model getting any information about the flaw from the function name.

```
void main()
{
    {
```

```
        char * data = (char *)calloc(100, sizeof(char));
        if (data == NULL)
        {
        }
        strcpy(data, "Body");
        printLine(data);
        if (data != NULL)
        {
            free(data);
        }
    }
}
```

We delete all newlines and tabs, replace them with spaces.

```
void f() { { char * data = (char *)calloc(100, sizeof(char)); if (data == NULL) {
} strcpy(data, "Body"); printLine(data); if (data != NULL) { free(data); } } }
```

After that, we delete all unnecessary spaces, before and after syntax. This includes parentheses, comma, mathematical operators (except for * because it could also appear in variable definitions).

```
void f(){{char * data=(char *)calloc(100,sizeof(char));if(data==NULL){}strcpy(
data,"Body");printLine(data);if(data!=NULL){free(data);}}}
```

We consider this to be the most compact form of a C/C++ function.

Sometimes the instance contains multiple functions, with a sink function or a helper function, we keep those functions, except changing the function names to simply `sink, helper` etc.

Finally, for word level models, we parse the above instance into list of words by using standard C/C++ syntax (including space) as anchors to break up the string. The final output of this example case is a list of 65 ”words”.

```
['void', ' ', 'f', '(', ')', '{', '{', 'char', ' ', '*', ' ', 'data', '=', '(',
'char', ' ', '*', ')', 'calloc', '(', '100', ',', 'sizeof', '(', 'char', ')', ')',
';', 'if', '(', 'data', '==', 'NULL', ')', '{', '}', 'strcpy', '(', 'data', ',',
'"', 'Body', '"', ')', ';', 'printLine', '(', 'data', ')', ';', 'if', '(',
'data', '!=', 'NULL', ')', '{', 'free', '(', 'data', ')', ';', '}', '}', '}']
```

We further add the beginning token <sos> and end token <eos> to the list, and pad the list using pad token <pad> until the length of the list reaches the target length. This length is 500 words for C/C++ dataset and 600 for Java dataset.

2 POWER AND RESOURCE OVERHEAD ESTIMATION

Here we provide a table that contains estimations of the time and power consumption for all different models we trained. The power estimation is an overestimate that may be larger than the real values: since the power consumption varies when running, it is hard to estimate exactly. For all methods listed, we use the computation resource of 1 GeForce GTX 1080 Ti graphic card, and 1 CPU for more than 95% of the time during training. According to tech specs provided by NVIDIA, the maximum GPU power consumption is 250W while the required system power consumption is 600 W. Therefore, we use a value

of 1kW for each hour of running for power consumption, as estimation. The time estimation is the average training time for each of the models for each fold in our four-fold cross validation.

Time, power and storage overheads for Different Models			
Model Type	Time Consumption (Hours)	Power Consumption (kW· h)	Model Parameters (millions)
Transformer	6.45	6.45	23.52
Pyr Transformer	3.25	3.25	26.67
GRU+Bahdanau Att	32.68	32.68	12.36
Pyr GRU+Bahdanau Att	20.81	20.81	11.08
GRU+Luong's general Att	13.42	13.42	12.36
Pyr GRU+Luong's general Att	7.57	7.57	11.08
GRU+Luong's dot Att	4.76	4.76	12.04
Pyr GRU+Luong's dot Att	2.03	2.03	10.76
GRU+Luong's concat Att	154.09	154.09	12.36
Pyr GRU+Luong's concat Att	117.94	117.94	11.08
